# Clustering of comorbid conditions among women who carry an *FMR1* premutation

**DOI:** 10.1038/s41436-019-0733-5

**Published:** 2020-01-03

**Authors:** Emily Graves Allen, Krista Charen, Heather S. Hipp, Lisa Shubeck, Ashima Amin, Weiya He, Jessica Ezzell Hunter, Stephanie L. Sherman

**Affiliations:** 1 Department of Human Genetics, Emory University School of Medicine, Atlanta, GA USA; 2 Department of Gynecology and Obstetrics, Emory University School of Medicine, Atlanta, GA USA; 3 Center for Health Research, Kaiser Permanente Northwest, Portland, OR USA

**Keywords:** *FMR1*, premutation, FXPOI, FXTAS, fragile X syndrome

## Abstract

**Purpose:**

Emerging evidence indicates that women who carry an *FMR1* premutation can experience complex health profiles beyond the two well-established premutation-associated disorders: fragile X–associated primary ovarian insufficiency (FXPOI, affects ~20–30% carriers) and fragile X–associated tremor–ataxia syndrome (FXTAS, affects ~6–15% carriers).

**Methods:**

To better understand premutation-associated health profiles, we collected self-reported medical histories on 355 carrier women.

**Results:**

Twenty-two health conditions were reported by at least 10% of women. Anxiety, depression, and headaches were reported by more than 30%. The number of comorbid conditions was significantly associated with body mass index (BMI) and history of smoking, but not age. Survival analysis indicated that women with FXPOI had an earlier age at onset for anxiety and osteoporosis than women without FXPOI. Cluster analysis identified eight clusters of women who reported similar patterns of comorbid conditions. The majority of carriers (63%) fell into three categories primarily defined by the presence of only a few conditions. Interestingly, a single cluster defined women with symptoms of FXTAS, and none of these women had FXPOI.

**Conclusion:**

Although some women with a premutation experience complex health outcomes, most carriers report only minimal comorbid conditions. Further, women with symptoms of FXTAS appear to be distinct from women with symptoms of FXPOI.

## INTRODUCTION

Carrying an *FMR1* premutation (PM) allele (55–199 CGG repeats) poses risks for varied health consequences, some of which are unique to women. First, a PM carried by women, but not those carried by men, can be transmitted to their offspring as an expanded full mutation (FM, >200 methylated repeats), leading to fragile X syndrome (FXS),^1^ the most common genetic form of intellectual and developmental disability (IDD) and of autism spectrum disorder.^2^ Second, women with a PM are at risk for fragile X–associated primary ovarian insufficiency (FXPOI), with 20–30% of women with a PM experiencing irregular or absent menstrual cycles due to ovarian insufficiency prior to age 40.^3^ Third, women and men with a PM are at risk for developing fragile X–associated tremor–ataxia syndrome (FXTAS), typically after age 60,^4,5^ although women have a lower absolute risk for FXTAS compared with men.^6^ Fourth, emerging reports suggest women are at higher risk for developing other health problems, including autoimmune disorders, chronic pain disorders, fibromyalgia, endocrine disorders, and mental health disorders.^7,8^ Whether these conditions are sex-specific manifestations of a PM or result from a stressful environment that is sometimes the consequence of carrying a PM is unknown.

Following the description of FXPOI in 1999,^9^ medical comorbidities related to FXPOI, such as osteoporosis and climacteric symptoms, were identified.^3,10^ More recently, a broader spectrum of disorders among women carrying a PM has emerged. Coffey et al.^11^ reported a significant increase in reporting of thyroid problems, hypertension, seizures, fibromyalgia, muscle pain, and symptoms related to FXTAS such as tremor, ataxia, and neuropathy in women with a PM. When this study sample was expanded, a significant increase in immune-mediated disorders among women with a PM was seen.^12^ Some of these medical conditions are reported more frequently among women with FXTAS compared with those without a FXTAS diagnosis.^7,11,12^

In addition to physical health conditions, mental health problems have also been noted. A national parent survey found an increase in reporting for anxiety (31%), depression (28%), and attention problems (14%) in their children with a PM (ages ≥6 years).^13^ A study by Hunter et al.^14^ reported a significant increase in mental health disorders (attention deficit–hyperactivity disorder [ADHD], anxiety, and depression) and learning disabilities among adult PM carriers compared with noncarriers. More recently, in a review of 20,000 electronic health records, Movaghar et al.^15^ identified several mental health diagnoses, i.e., agoraphobia, social phobia, and anxiety disorder, as occurring more frequently in PM women.

The cause of these varied conditions is currently unknown. Increased comorbidity could be due to the biological impact of the PM itself. Alternatively, it could be the result of the many challenges facing women who carry the PM. First, caring for a child with IDD leads to higher levels of maternal stress relative to mothers of typically developing children due to the unique psychosocial, financial, and physical challenges.^16–18^ Elevated maternal stress can decrease maternal quality of life by elevating rates of depression and anxiety.^17,19–22^ Also, most women with FXPOI struggle with infertility, which affects quality of life and overall health.^23,24^ Nonetheless, Hagerman et al. recently termed this group of PM-associated conditions “FXAND: fragile X–associated neuropsychiatric disorders.”^25^

The overall aim of this work is to identify whether there is a subgroup of women who are more vulnerable to complex medical issues or whether there is a global impact of the PM. To do so, we collected self-report health and reproductive histories on 355 women with a PM. We hypothesized that women with FXPOI may be more vulnerable to these comorbid conditions; however, the frequency of conditions did not differ—only an earlier age of onset for anxiety and, as expected, osteoporosis, among women with FXPOI was found. We then used cluster analysis to further identify subgroups using the 22 conditions endorsed by >10% of PM women. Demographic, environmental, and reproductive variables were used to further characterize each cluster.

## MATERIALS AND METHODS

### Study population

Protocols and consent forms were approved by Emory University Institutional Review Board, and informed consent was obtained from all participants. Participants were identified from previous FXS research projects at Emory, recruitment at scientific conferences, and through collaborations with other research groups. Once identified, additional family members were screened for eligibility without respect to phenotype. Eligibility was based on PM carrier status, age, and sex. A blood or saliva sample was collected, and each participant completed a reproductive and health history questionnaire. Data included general demographics (e.g., age at interview, date of birth, race/ethnicity), lifestyle factors that might affect overall health (e.g., smoking, body mass index [BMI]), reproductive history (e.g., menstrual history, reason for cessation of menses, pregnancy history), and general medical history. For the medical history, participants reported the presence or absence of various conditions by indicating 0: “I do not have this condition,” 1: “I think I have this but have not been diagnosed by a medical professional,” or 2: “I have been diagnosed with this by a medical professional”. If option 2 was chosen, age at diagnosis was asked. Sixty-three conditions were queried on the medical history questionnaire. Any condition reported by >10% of all women carrying a PM was included in further data analyses.

The reproductive history was used to determine whether a woman was still cycling or why her periods had stopped. Women were defined as having FXPOI if their age at natural menopause (AAM) was < age 40, excluding those with iatrogenic (e.g., hysterectomy/oophorectomy) or alternative causes of menses cessation. Women were classified as having FXPOI if they had absent menses for at least 4–6 months along with menopausal-level follicle-stimulating hormone.^26^ Women who had menopause or were still having menstrual cycles at age 40 or later were classified as not having FXPOI. For some women, a FXPOI assignment could not be made (e.g., women who were still cycling but younger than age 40, or women who had surgery, such as a hysterectomy, before age 40).

### Laboratory methods

DNA was extracted from biological samples using Qiagen Qiamp DNA Blood Mini Kit, Gentra Puregene extraction kit, or prepIT-L2P protocol from Oragene.

FRAXA CGG repeat numbers were determined by a fluorescent sequencer method.^27^ For females with only one allele, a second polymerase chain reaction (PCR) protocol was used.^28^ The PCRs for FRAXA consisted of 1X PCR Buffer (Gibco/BRL), 10% dimethyl sulfoxide (DMSO), 370 µM deazaG, 500 µM d(ACT), 0.3 µM each primer, 15 ng T4 gene 32, and 1.05 U Roche Expand Long *Taq*. Primers for the FMR1 gene were C: 5′GCTCAGCTCCGTTTCGGTTTCACTTCCGGT3′, and F: 5′AGCCCCGCACTTCCACCAGCTCCTCCA3′.^29^

### Statistical analysis

Standard statistical methods were used including *t*-tests for continuous measures and chi-square analyses for categorical variables. For comparisons of conditions by FXPOI status or CGG repeat size, logistic regression models were used and adjusted for age at interview. For these analyses, we initially combined options 1 and 2 as a positive endorsement because, for some of the conditions, e.g., anxiety or depression, the presence of symptoms may negatively impact the participant’s willingness to seek a medical diagnosis. However, as a sensitivity analysis, we also tested all models using only option 2 as a positive endorsement. To compare the age at onset of conditions between FXPOI and non-FXPOI groups, survival analyses were used. For this analysis, women who had been diagnosed by a medical professional (option 2) were included as those with the “event,” because only these women were asked about an age of onset. For these models, the age of onset was used as the “event” age. Women who did not report a diagnosis by a medical professional (options 0 and 1) were censored from the analysis at their age of interview. To ensure there was not a bias in including the women who endorsed option 1, all survival models were also run without these women included as a sensitivity analysis. Linear regression models were used to test for associations with the total number of conditions reported.

For all analyses of the reported conditions, a Bonferroni correction was used to assess significant differences. Because 22 total conditions were analyzed, a conservative *p* value of 0.002 was used as the threshold for significance.

To perform cluster analysis, a data set was created that only included the participant ID and the binary variable for each of the 22 conditions (participants who selected option 1 or 2 were classified as “1,” and participants who reported option 0 were classified as “0”). Three women were dropped from analysis due to missing data for at least one of the included conditions. Cluster analysis using Ward’s minimum-variance clustering method was used to group participants who reported similar combinations of conditions. Ward’s method maximized outcome statistical measures including *R*^2^, the pseudo-F statistic, and the root mean square distance between observations. We tested multiple numbers of clusters in our analyses, and based on the statistical parameters and an evaluation of outcomes, a model with eight clusters was chosen. Additional information about each *n*-cluster model is shown in Supplementary Fig. 1. Descriptive names were assigned to the eight clusters to summarize the characteristics that distinguished them. These assigned names are only used for reference purposes throughout this paper; they have no meaning in terms of medical diagnoses.

Analysis of variance (ANOVA) models were used to compare means of continuous measures (e.g., BMI) between clusters and significant differences between groups were determined using Tukey’s post hoc analysis to control for multiple testing. Logistic regression models were used to test for differences between clusters for binary variables (e.g., FXPOI).

All analyses were done using SAS 9.4.

## RESULTS

Table 1 presents basic demographic information for our study population. In total, 355 women with a PM completed the reproductive and medical history questionnaire and provided a biological sample for *FMR1* genotyping. Because we were interested in whether any comorbid conditions were seen more frequently in women with FXPOI, we compared the demographic information for women with FXPOI (AAM <age 40) and women without FXPOI. A total of 87 and 168 women, respectively, were included in these groups. There were no statistically significant differences in race, BMI, history of smoking, number of children with FXS, and number of comorbid conditions reported by these two groups. Statistically significant differences among other demographic variables indicated that women with FXPOI were younger, had a lower average number of children, and more often were not satisfied with their number of children. In addition, although the mean CGG repeat size was not statistically different, the distribution of repeat size categories differed: those with FXPOI were more likely to have 80–100 repeats.

**Table 1:** Demographic, environmental, and reproductive information on study participants.

	All PM	FXPOI	No FXPOI
*N*	355	87	168
Age at interview	47.4 ± 12.5	46.2 ± 10.6	53.5 ± 8.8^a^
Mean ± SD (min–max)	(19–93)	(26–72)	(37–80)
Race			
% White	90.4	93.1	88.7
% Black	3.7	2.3	4.8
% Hispanic	3.9	2.3	4.8
% Other	2.0	2.3	1.7
Body mass index (BMI)	27.4 ± 6.8 (17.4–63.4)	27.6 ± 6.8 (17.5–49.9)	28.0 ± 6.4 (18.2–55.0)
% Ever smoked	27.7	24.1	29.3
Number of children	1.7 ± 1.3	1.6 ± 1.2	2.0 ± 1.1^b^
Mean ± SD (Min–max)	(0–8)	(0–5)	(0–6)
Number of children with FXS	0.8 ± 0.8	0.7 ± 0.8	0.8 ± 0.7
Mean ± SD (Min–max)	(0–3)	(0–2)	(0–3)
% Satisfied with number of children	78.7	65.5	88.1^c^
Number of conditions: mean ± SD; median; mode (min–max)	4.0 ± 3.5; 3; 1 (0–16)	4.4 ± 4.0; 3; 1 (0–16)	3.9 ± 3.4; 3; 0 (0–15)
Repeat size			
Mean ± SD (Min–max)	91.9 ± 19.4 (56–190)	89.6 ± 14.2 (56–140)	91.7 ± 20.7 (56–190)
% 55–79	25.0%	19.8%	28.1%^d^
% 80–100	49.4%	61.6%	44.5%
% 101–200	25.6%	18.6%	27.4%

*FXPOI* fragile X–associated primary ovarian insufficiency, *FXS* fragile X syndrome, *PM* premutation.

^a^*p* < 0.0001 using a *t*-test to compare means among women with and without FXPOI.

^b^*p* < 0.05 using a *t*-test to compare means among women with and without FXPOI.

^c^*p* *<* 0.0001 using chi-square analysis to compare frequencies among women with and without FXPOI.

^d^*p* < 0.05 using chi-square analysis to compare frequencies among women with and without FXPOI.

We next investigated the self-reported medical history. Of the 63 conditions listed in the medical history questionnaire, 22 conditions were positively endorsed by >10% of all women with a PM (Table 2). The frequencies of the remaining conditions are in Supplemental Table 1. The most frequently reported conditions were anxiety and depression, followed by migraine and tension headaches (Table 2).

**Table 2:** Frequency of reported conditions for all women with a PM, those with FXPOI, and those without FXPOI, listed in order of frequency reported by all PM women.

	All PM (*N* = 355)		FXPOI (*N* = 87)		No FXPOI (*N* = 168)		*P* values for models comparing FXPOI and no FXPOI^c^		
	% Total	% Option 1^a^ /% option 2^b^	% Total	% Option 1^a^ /% option 2^b^	% Total	% Option 1^a^ /% option 2^b^	Model 1: logistic regression	Model 2: logistic regression	Survival analysis
Anxiety	37.8%	15.2% / 22.5%	44.8%	12.6% / 32.2%	30.4%	12.5% / 17.9%	0.161	0.150	**0.001**
Depression	35.5%	8.4% / 27.0%	33.3%	2.3% / 31.0%	35.1%	10.1% / 25.0%	0.412	0.541	0.044
Migraine headaches	33.2%	12.4% / 20.8%	33.3%	9.2% / 24.1%	26.8%	8.3% / 18.4%	0.809	0.485	0.087
Tension headaches	31.5%	21.7% / 9.9%	31.0%	17.2% / 13.8%	28.6%	22.0% / 6.5%	0.671	0.098	0.026
Sleep problems	28.7%	20.8% / 7.9%	34.5%	26.4% / 8.0%	30.4%	21.4% / 8.9%	0.249	0.914	0.608
Peripheral neuropathy	20.3%	14.7% / 5.6%	22.1%	16.3% / 5.8%	21.4%	14.3% / 7.1%	0.802	0.811	0.899
IBS	19.7%	8.2% / 11.5%	19.5%	6.9% / 12.6%	17.9%	8.3% / 9.5%	0.613	0.710	0.201
Osteoporosis	19.1%	1.4% / 17.7%	26.4%	0% / 26.4%	20.8%	1.8% / 19.0%	0.017	0.056	**0.001**
Hypothyroidism	17.5%	3.1% / 14.4%	23.0%	2.3% / 20.7%	17.9%	3.0% / 14.9%	0.221	0.114	0.033
Hypertension	16.9%	0.6% / 16.3%	10.3%	1.1% / 9.2%	23.8%	0% / 23.8%	0.130	0.074	0.170
RLS	15.2%	11.3% / 3.9%	12.6%	9.2% / 3.4%	14.9%	11.3% / 3.6%	0.895	0.544	0.381
Ataxia	13.5%	9.9% / 3.7%	9.2%	5.7% / 3.4%	15.5%	11.3% / 4.2%	0.799	0.578	0.562
Sleep apnea	13.0%	5.6% / 7.3%	14.9%	6.9% / 8.0%	16.7%	6.5% / 10.1%	0.870	0.719	0.460
Chronic muscle pain	11.9%	7.3% / 4.5%	14.9%	5.7% / 9.2%	10.1%	7.1% / 3.0%	0.125	0.027	0.025
Social phobia	11.8%	10.7% / 1.1%	20.7%	18.4% / 2.3%	10.7%	9.5% / 1.2%	0.025	0.462	0.370
Fibromyalgia	11.5%	4.5% / 7.0%	16.1%	3.4% / 12.6%	9.5%	4.8% / 4.8%	0.100	0.019	0.005
CFS	11.3%	9.0% / 2.2%	14.9%	11.5% / 3.4%	8.3%	6.5% / 1.8%	0.083	0.382	0.201
TMJ	11.3%	1.4% / 9.9%	16.0%	4.6% / 11.5%	10.7%	0% / 10.7%	0.170	0.846	0.473
OCD	10.7%	8.4% / 2.2%	10.3%	5.7% / 4.6%	11.9%	9.5% / 2.4%	0.288	0.980	0.228
ADHD	10.7%	7.6% / 3.1%	12.6%	10.3% / 2.3%	10.1%	6.5% / 3.6%	0.974	0.289	0.736
LD	10.4%	7.6% / 2.8%	6.9%	5.7% / 1.1%	10.7%	8.3% / 2.4%	0.347	0.124	0.589
Tremor	10.1%	7.0% / 3.1%	8.0%	5.7% / 2.3%	11.9%	8.3% / 3.6%	0.811	0.823	0.806

Model 1: endorsement of either option 1 or option 2 was used to define affected individuals. Model 2: endorsement of option 2 only is used to define affected individuals. Endorsement of option 1 is grouped with option 0 as the unaffected population.

*ADHD* attention deficit–hyperactivity disorder, *CFS* chronic fatigue syndrome, *IBS* irritable bowel syndrome, *LD* learning disability, *OCD* obsessive compulsive disorder, *RLS* restless leg syndrome, *TMJ* temporomandibular joint dysfunction.

^a^% of subjects who selected “I think I have this but have not been diagnosed by a medical professional.”

^b^% of subjects who selected “I have been diagnosed with this by a medical professional.”

^c^Bonferroni-adjusted statistical significance *p* < 0.002 are bolded; Marginally significant models where significance was between 0.001 and 0.05 are underlined.

We investigated associations for each condition with repeat size using logistic regression. Because several conditions are associated with age, we adjusted these models for age at interview (Supplementary Table 2, model 1). In our models, we tested for both a linear relationship with repeat size (Supplementary Table 2, model 2) and the nonlinear relationship seen with risk for FXPOI (Supplementary Table 2, model 3). Of the 22 conditions, only peripheral neuropathy showed an association with repeat size (Supplementary Table 2; *p* = 0.001).

We then compared the frequency of each condition in women with FXPOI to women without FXPOI. When options 1 and 2 were combined (model 1, Table 2), osteoporosis and social phobia showed a marginally significant difference in a logistic regression model adjusted for age at interview (*p* = 0.017 and *p* = 0.025, respectively). When only women who endorsed option 2 were included as the affected individuals in logistic regression models adjusted for age at interview (model 2, Table 2), FXPOI women had a marginally significant increased reporting of diagnoses of chronic muscle pain and fibromyalgia (*p* = 0.027 and *p* = 0.019, respectively). However, none of these results met our Bonferroni threshold of 0.002 for significance. Comparing these women using survival analysis revealed a significantly earlier age of onset for osteoporosis and anxiety among women with FXPOI (*p* = 0.001). Other marginally significant findings for an earlier age of onset included fibromyalgia, chronic muscle pain, tension headaches, hypothyroidism, and depression (Table 2). In sensitivity analyses that did not include the responses from women who selected option 1, all conclusions were the same.

To summarize the overall health condition of each woman, we summed the number of conditions reported per woman for the 22 conditions and examined the frequency distribution (Supplementary Fig. 2). About half of the women (47%) reported four or more conditions. We then asked whether the number of endorsed conditions was associated with demographic, environmental, or reproductive variables (Table 3). Both BMI and history of smoking (*p* < 0.0001) were associated with the number of conditions reported.

**Table 3:** Associations of demographic, environmental, and reproductive variables with number of conditions reported.

	Sample size in model	β coefficient	*R*^2^	*p* value^a^
Mean age	354	0.026	0.01	0.0810
BMI	352	0.128	0.06	**<0.0001**
Ever smoked (y/n)	354	1.779	0.05	**<0.0001**
Number of children	355	0.371	0.02	0.0120
Number of children with FXS	348	0.380	0.01	0.1202
Repeat size (continuous)	348	0.011	0.00	0.2624
FXPOI (y/n)	255	0.416	0.00	0.3841
AAM (age of onset)	198	−0.051	0.01	0.0933

*AAM* age at menopause, *BMI* body mass index, *FXPOI* fragile X–associated primary ovarian insufficiency.

^a^Bonferroni-adjusted statistical significance *p* < 0.002 are bolded; Marginally significant models where significance was between 0.001 and 0.05 are underlined.

Cluster analysis using the 22 conditions from Table 2 identified eight clusters of women based on their endorsement of each condition. This final model of eight clusters explained 31% of the variance in reporting of health conditions. We characterized each cluster based on frequencies of reported conditions within each cluster (Table 4) and other associated descriptive variables (Table 5). Three clusters were designated as FXPOI because of greater than the expected frequency (~20%) of women with FXPOI in each of the clusters. We assigned a descriptive label for each of the eight clusters as follows: (1) minimal health problems, (2) headaches, (3) sleep problems, (4) mental health problems, (5) FXPOI with minimal health problems, (6) FXPOI with mental health problems, (7) FXPOI with complex profiles, and (8) FXTAS symptoms. Table 4 shows a heat map based on the proportion of women reporting of each of the 22 conditions within each cluster. Table 5 shows demographic and descriptive statistics for each of the clusters. Characteristics and findings of interest for each of the clusters are summarized below.

**Table 4:**
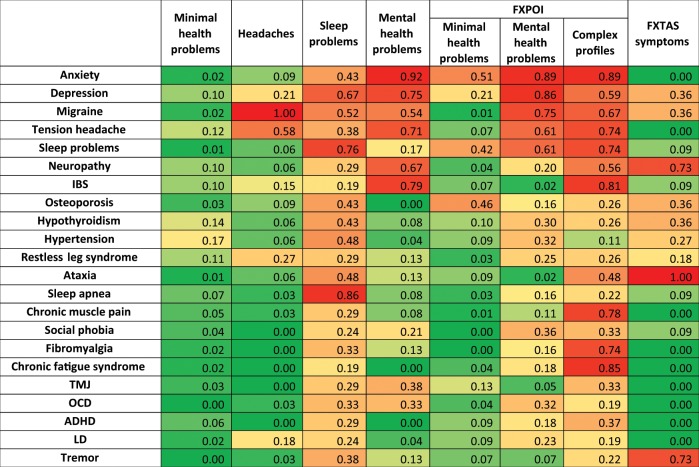
Heat map showing frequencies of reported conditions within each cluster.

Color scale: red indicates increased reporting (either option 1 or 2) of condition within the cluster and green represents decreased reporting of conditions within the cluster.

*ADHD* attention deficit–hyperactivity disorder, *CFS* chronic fatigue syndrome, *FXTAS* fragile X–associated tremor–ataxia syndrome, *IBS* irritable bowel syndrome, *LD* learning disability, *OCD* obsessive compulsive disorder, *RLS* restless leg syndrome, *TMJ* temporomandibular joint dysfunction.

**Table 5:**
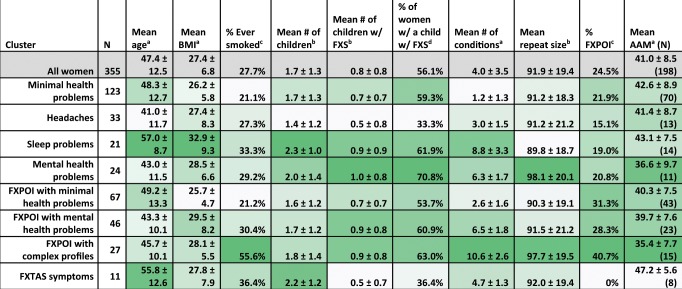
Demographic, environmental, and reproductive information on study participants in each of the eight clusters.

Color scale: green indicates ranking of clusters within each column relative to other clusters. Color does not indicate significance. The information for all women in the study sample is provided for comparison.

*AAM* age at menopause, *BMI* body mass index, *FXPOI* fragile X–associated primary ovarian insufficiency, *FXS* fragile X syndrome, *FXTAS* fragile X–associated tremor–ataxia syndrome.

^a^Significant differences were seen between clusters in an analysis of variance (ANOVA) model (See Supplementary Fig. 3).

^b^No significant differences were seen between clusters in ANOVA models.

^c^*p* < 0.05 for chi-square analysis.

^d^Not significant in chi-square analysis.

### Minimal health problems cluster

The largest cluster was the minimal health problems cluster with 123 women. Significantly fewer health conditions were reported by women in this cluster compared with any other cluster (Table 5; Supplementary Fig. 3C). We hypothesized that women in this cluster might be younger than those in the other clusters, as many conditions were age-dependent, but this was not the case (Table 5; Supplementary Fig. 3A). Consistent with our findings of features associated with the number of health conditions per woman (Table 3), mean BMI (Table 5; Supplementary Fig. 3B) and history of smoking (Table 5) were found to be relatively lower for women in this cluster.

### Headaches cluster

All 33 women in this cluster endorsed migraine headaches and 58% endorsed tension headaches (Table 4). They were younger than those in other clusters (Table 5; Supplementary Fig. 3A) and reported few additional conditions (Tables 4, 5).

### Sleep problems cluster

The sleep problems cluster includes 21 women and significantly differed from all other clusters for the number of conditions reported; on average, the women reported 8.8 comorbid conditions (Table 5; Supplementary Fig. 3C). Table 4 shows the clear impact on overall health for women in this cluster. Associated characteristics included increased age at interview and increased BMI (Table 5; Supplementary Fig. 3A, B).

### Mental health problems cluster

This cluster includes 24 women and is a relatively younger group with the highest proportion of women with a child with FXS (Table 5). Overall, the mental health problems cluster looks similar to the FXPOI with mental health problems cluster for conditions reported and associated demographics. Factors that distinguish these two clusters are related to FXPOI, such as osteoporosis (Table 4) and a lower frequency of FXPOI (Table 5). Other comorbid conditions that distinguish this cluster from the FXPOI cluster are the higher frequency of neuropathy and IBS and the lower frequencies of hypothyroidism, hypertension, and chronic fatigue syndrome (Table 4).

### FXPOI with minimal health problems cluster

The three FXPOI clusters were designated as FXPOI because there is greater than the expected frequency of women with FXPOI in each. Distinguishing characteristics of the 67 women in this cluster include low BMI, >50% have a child with FXS, and a low number of conditions are reported (Table 5).

### FXPOI with mental health problems cluster

The 46 women in this cluster have a higher proportion of women with a child with FXS (Table 5). The average number of conditions reported by women in this cluster is 6.5 (Table 5). More than 60% of women endorsed anxiety, depression, migraine headaches, tension headaches, and sleep problems (Table 4).

### FXPOI with complex profiles cluster

This cluster includes 27 women, and these women report the highest number of co-occurring conditions (Table 5; Supplementary Fig. 3C). Age at interview was not increased in this group, the mean AAM is lower than all other groups, and the percentage with FXPOI is highest (Table 5; Supplementary Fig. 3D). Importantly, this cluster also reported the highest history of smoking (Table 5).

### FXTAS symptoms cluster

The final cluster included 11 women and was clearly delineated as the FXTAS cluster, with all women reporting ataxia and more than 70% reporting tremor and neuropathy with few other conditions (Table 4). There were no women with FXPOI within this cluster, and the average age at menopause within the group was the highest of all other groups (Table 5; Supplementary Fig. 3D). Additional information including FXTAS diagnostic status is shown in Supplementary Table 3.

## DISCUSSION

Of the 22 conditions reported by at least 10% of the women included in this study who carry a PM, the most frequently reported conditions were anxiety, depression, headaches, and sleep problems. These descriptive findings suggested that there were distinct classes of women with respect to their health: many women reported few or no comorbid conditions, whereas other women had complicated health histories with as many as 16 reported conditions. The logical next step was to identify whether these conditions clustered across all women with a PM. Combining the results from the descriptive, survival, and cluster analyses, we made the following observations:

1. *The majority of women with a PM report few comorbid conditions*. The majority of women (>60%) fall into the minimal health problems, headaches, and FXPOI with minimal health problems clusters, where few conditions other than the defining characteristics (e.g., headaches in the headaches cluster) were reported.
2. *Several of the previously reported conditions reported in the literature are not reported by >10% of our population*. Previous studies have identified an increased reporting or some of the queried conditions from our medical history, including seizures and autoimmune disorders (Supplementary Table 1).^11,12^ Specific autoimmune disorders were endorsed, but at low frequency in this sample. In future studies, it may be useful to combine those with similar etiologies, where possible.
3. *An association between having a child with FXS was seen in clusters with high reporting of anxiety and depression*. The four clusters with the highest percentage of women with a child with FXS (>60%) also have high reporting of anxiety and depression.
4. *Age at interview was not associated with the number of conditions*. Age at interview was not significantly associated with the number of conditions reported in the overall data set. Further, the clusters identified with the most complex health histories did not have an increased age at interview.
5. *Risk of FXTAS symptoms appears to be distinct from the risk for FXPOI*. Interestingly, of the 11 women who clustered into the FXTAS symptoms cluster, none met the definition for FXPOI, and this cluster of women has the highest average age at menopause. This is consistent with other studies that found that the risk for FXPOI is not increased among women with symptoms of FXTAS compared with controls.^11,30^

Our original hypothesis was that many of these comorbid conditions would be associated with a diagnosis of FXPOI; however, we did not find this to be true. Based on our results, it seems unlikely that the molecular mechanism related to complex medical histories is the same as the nonlinear association with CGG repeat size seen with the risk for FXPOI.^3,31,32^ For FXTAS, current data support two non–mutually exclusive molecular pathogenesis mechanisms: transcribed PM alleles carry expanded CGG repeats that can be found in RNA foci^33^ and/or inclusions,^34^ and the PM CGG repeat expansion induces RAN translation within the 5′ UTR of *FMR1* messenger RNA (mRNA), producing polypeptides that may be toxic.^35^ In our data, the size of the PM was not associated with the endorsement of complex health histories (Tables 3, 5, and Supplementary Table 2). Clearly, follow-up molecular studies, such as genome sequencing (GS) data to identify modifying factors and/or metabolomics to identify alterations in metabolic profiles, are the necessary next steps to identify factors that put particular women with a PM at risk.

There are several limitations to this research. Most notably, these data are based on self-report. The population that has participated in our research may have some biases: women with more complicated health histories may have greater motivation to participate in research, or conversely, women with minimal health conditions may have more time and energy for participating in research. Additionally, many of the families that participate in our research come to our attention at conferences, which potentially biases our sample toward families of higher socioeconomic status. Our goal in this work was to understand the heterogeneity of health conditions among women with a PM; however, data from women who do not carry a PM would help establish whether the increased frequency of conditions and clustering is unique to carriers. Also of note, our questionnaire was designed to ask about lifetime occurrence of these conditions. We were not able to distinguish current diagnoses from lifetime diagnoses. Lastly, our use of cluster analysis and choice of the eight-cluster model may not provide the optimal model. However, each solution did indicate significant heterogeneity of health profiles among premutation carriers.

There are also several positive attributes to the study design: this is not a clinic-based population and therefore not selected for existing health conditions for which women were seeking medical care. In addition, all aspects of the project could be completed through the mail or online, eliminating any socioeconomic barriers such as childcare needs or travel or barriers related to mental health problems that potentially reduce the ability to interact directly with a study team.

In summary, as has been seen in numerous previous reports,^7,8,11,12,14,15,25^ we have confirmed a high reporting of numerous health conditions among the 355 women who carry a PM. Many of these diagnoses are similar to those identified in an analysis electronic medical records of an unbiased sample of women with a PM,^15^ including sleep apnea, gait problems, and mental health diagnoses. In our data set, both an elevated BMI and history of smoking showed a positive correlation with the number of conditions reported, indicating the importance of environmental factors beyond the susceptibility of carrying a PM allele. Although many conditions were reported, we need to emphasize that the majority (>60%) of women with a PM reported few or no health conditions. Overall, there was significant heterogeneity with respect to women’s global health conditions.

Future studies are needed to understand the basis of this heterogeneity. Importantly, the final model explained only 31% of the variation in the health histories. Examination of other possible variables, including maternal stress, medical triggers, environmental exposures and genetic modifiers, will be important next steps. The finding that the symptoms of FXTAS tended to cluster separately from other groups requires further investigation to determine whether this group can be better defined with respect to etiology and associated risk factors and how they may differ from the other PM-associated conditions. Lastly, we emphasize that results from our cluster analyses cannot be used for medical purposes; i.e., they do not define diagnostic subgroups. Instead, they provide the impetus for further research into understanding of the manifestation of the PM.

## Supplementary information

Supplementary Figures

Supplementary Figure Legends

Supplementary Tables
