# Supplementary material for: Clustering of comorbid conditions among women who carry an FMR1 premutation
Source: Genet Med. 2020 Jan 3;22(4):758–66. doi: 10.1038/s41436-019-0733-5 (PMC7118023; doi:10.1038/s41436-019-0733-5)
Supplement: Supplementary file 1 — Supplementary Figures [file 41436_2019_733_MOESM1_ESM.pptx]

## Slide 1
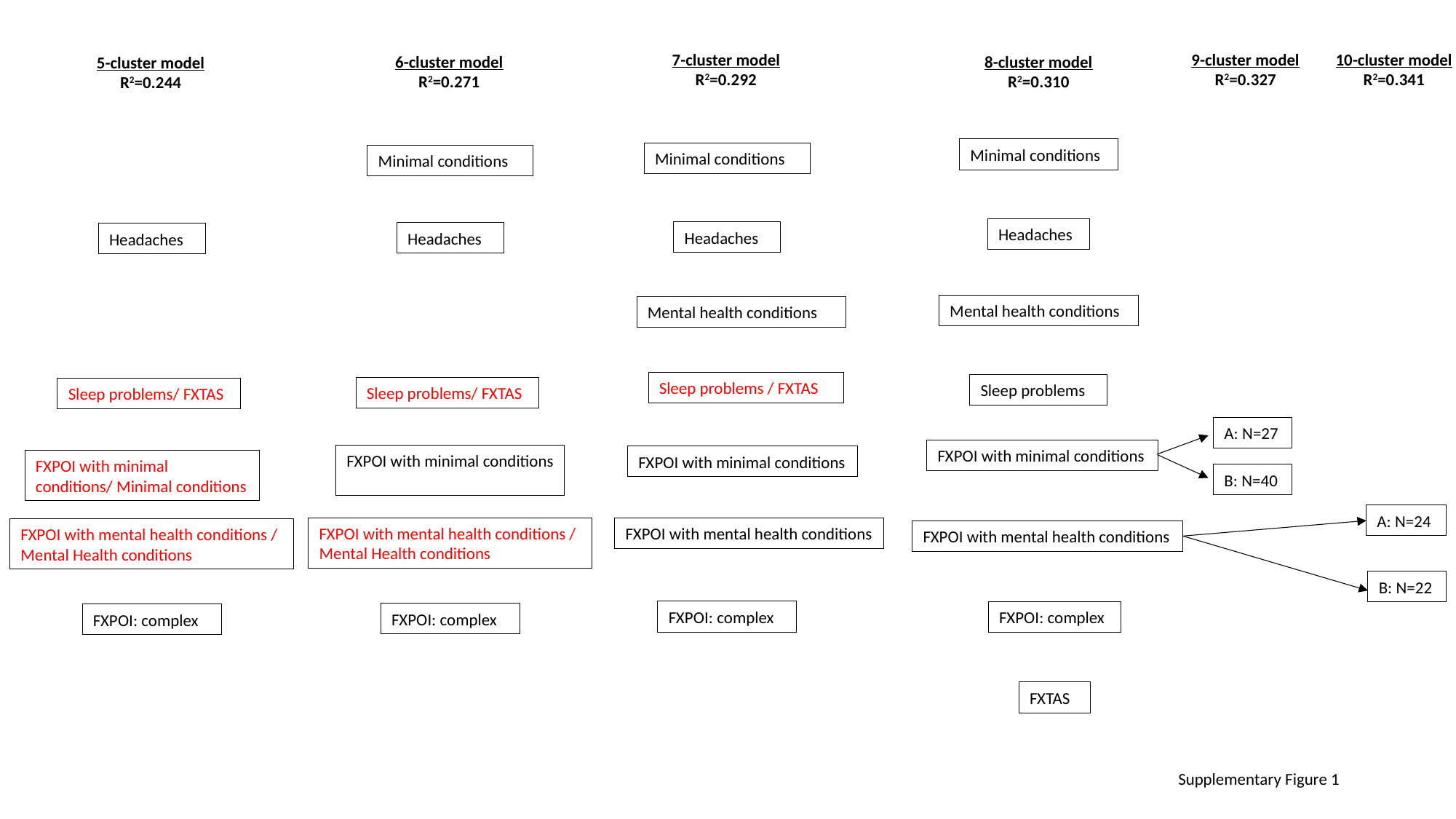

7-cluster model
R2=0.292
Minimal conditions
Headaches
Mental health conditions
Sleep problems / FXTAS
FXPOI with minimal conditions
FXPOI with mental health conditions
FXPOI: complex
9-cluster model
R2=0.327
10-cluster model
R2=0.341
6-cluster model
R2=0.271
Minimal conditions
Headaches
Sleep problems/ FXTAS
FXPOI with minimal conditions
FXPOI with mental health conditions / Mental Health conditions
FXPOI: complex
8-cluster model
R2=0.310
Minimal conditions
Headaches
Mental health conditions
Sleep problems
FXPOI with minimal conditions
FXPOI: complex
FXTAS
5-cluster model
R2=0.244
Headaches
Sleep problems/ FXTAS
FXPOI with minimal conditions/ Minimal conditions
FXPOI with mental health conditions / Mental Health conditions
FXPOI: complex
A: N=27
B: N=40
A: N=24
FXPOI with mental health conditions
B: N=22
Supplementary Figure 1

## Slide 2
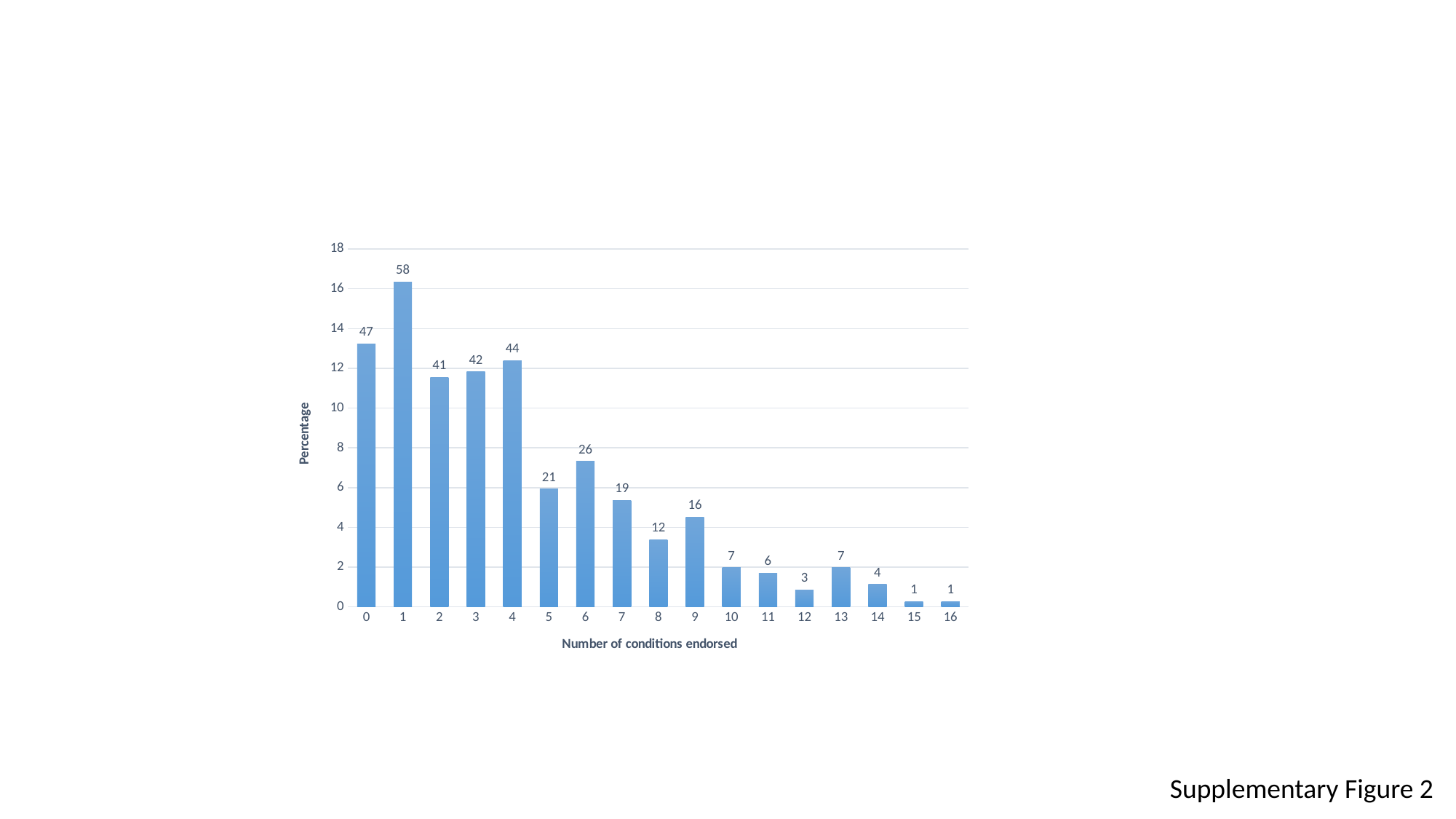

### Chart
| Category | |
|---|---|
| 0 | 13.24 |
| 1 | 16.34 |
| 2 | 11.55 |
| 3 | 11.83 |
| 4 | 12.39 |
| 5 | 5.92 |
| 6 | 7.32 |
| 7 | 5.35 |
| 8 | 3.38 |
| 9 | 4.51 |
| 10 | 1.97 |
| 11 | 1.69 |
| 12 | 0.85 |
| 13 | 1.97 |
| 14 | 1.13 |
| 15 | 0.28 |
| 16 | 0.28 |Supplementary Figure 2

## Slide 3
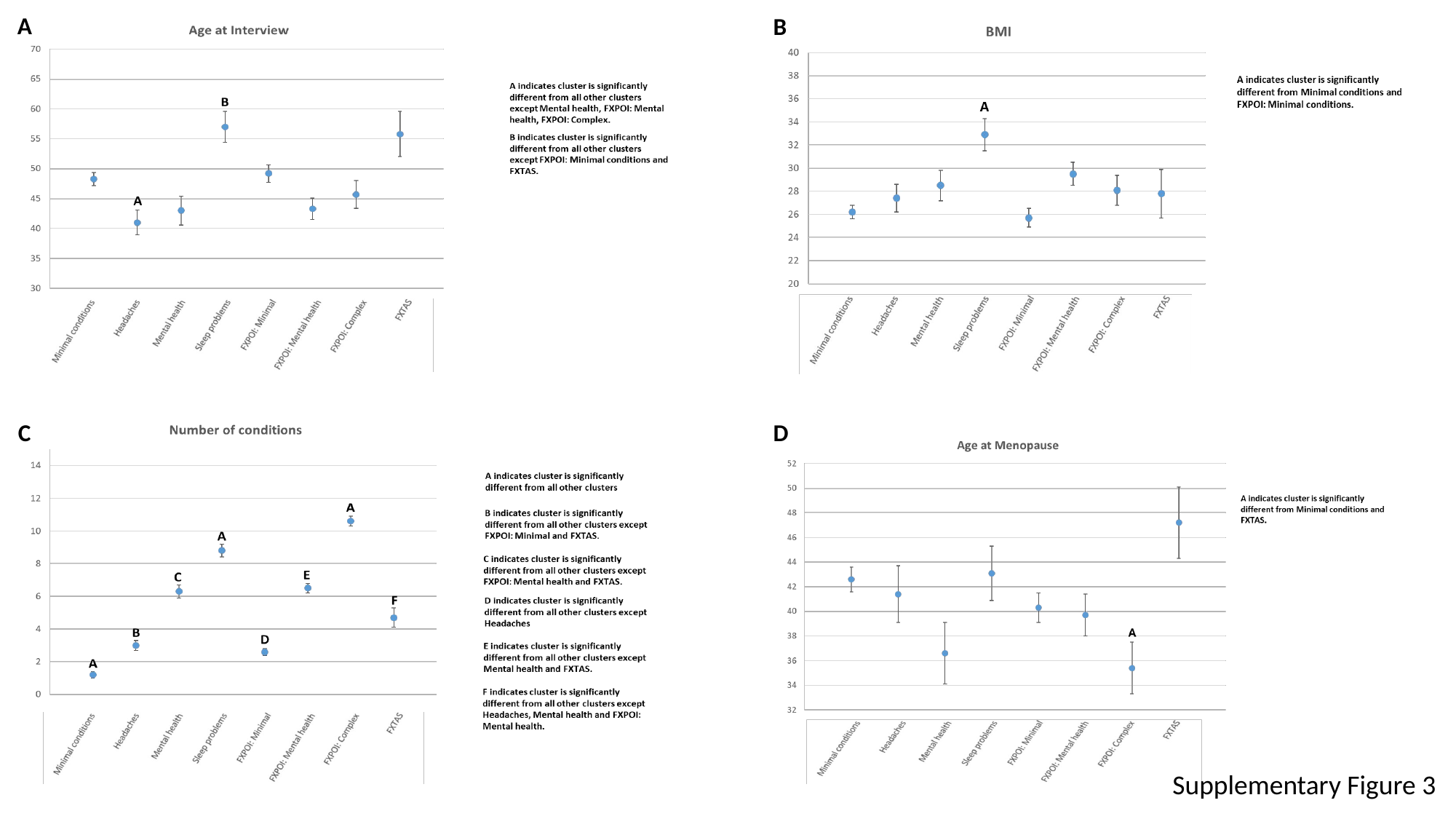

A
B
C
D
Supplementary Figure 3
