# Supplementary material for: Clustering of comorbid conditions among women who carry an FMR1 premutation
Source: Genet Med. 2020 Jan 3;22(4):758–66. doi: 10.1038/s41436-019-0733-5 (PMC7118023; doi:10.1038/s41436-019-0733-5)
Supplement: Supplementary file 2 — Supplementary Figure Legends [file 41436_2019_733_MOESM2_ESM.docx]

Supplementary Figure Legends

Figure 1. Cluster analysis selection of final model. Based on the sample size and our goal to efficently summarize the dataset, we examined models with five to ten clusters. R^2^ values are shown for each model, indicating the proportion of variance in health profiles explained by the number of clusters in each model. Models with fewer than eight clusters did not fully distinguish groups. The clusters shown in red for 5- to 7-cluster models were split into separate clusters in the 8-cluster model. For example, in models with 5 to 7 clusters, one group included two eventual clusters in the 8-cluster model that we labeled as Sleep Problems and FXTAS. In the 6-cluster model, the two Mental health conditions clusters were grouped together, and in the 5-cluster model, the two clusters with Minimal health conditions clustered together. Models with nine or ten clusters further divided two of our existing clusters; however, these clusters gave minimal additional information beyond the final 8-cluster model that was chosen.

Figure 2. Histogram of number of health conditions reported by premutation women.

Figure 3. Significant differences between clusters for age at interview, BMI, number of conditions, and age at menopause. Tukeys post hoc comparisons were used in each ANOVA model to determine significant pairwise differences for measures of age at time of interview (A), BMI (B), number of conditions reported (C), and age at natural menopause (D). All significant differences are indicated on the graph.
