# Supplementary material for: Clustering of comorbid conditions among women who carry an FMR1 premutation
Source: Genet Med. 2020 Jan 3;22(4):758–66. doi: 10.1038/s41436-019-0733-5 (PMC7118023; doi:10.1038/s41436-019-0733-5)
Supplement: Supplementary file 3 — Supplementary Tables [file 41436_2019_733_MOESM3_ESM.docx]

| Supplementary Table 1. Conditions that were not reported by more than 10% of PM carriers | | |
| --- | --- | --- |
| Condition | % think they have this (Option 1) | % diagnosed by a medical professional (Option 2) |
| Alcoholism | 1.40% | 0.80% |
| Autism spectrum disorder | 0.30% | 0% |
| Brain Bleed | 0.30% | 0.80% |
| Brain Injury | 0% | 1.70% |
| Cancer | 0% | 3.70% |
| Cerebral Palsy | 0% | 0% |
| Drug addiction | 0.30% | 0.30% |
| Female urethral syndrome/ interstitial cystitis | 1.70% | 3.70% |
| Hyperthyroidism | 1.10% | 3.10% |
| Lupus | 0.30% | 1.10% |
| Meningitis or Encephalitis | 0% | 1.10% |
| Mitral valve prolapse | 0% | 6.50% |
| Multiple chemical sensitivity | 2.20% | 0.60% |
| Multiple Sclerosis | 0.80% | 0.60% |
| Muscular Dystrophy | 0% | 0.30% |
| Myofascial pain syndrome | 1.70% | 0.80% |
| Myotonic Dystrophy | 0% | 0.60% |
| Optic Neuritis | 0.60% | 0.80% |
| Other autoimmune disorder | 0.80% | 3.90% |
| Other endocrine disorder | 0.60% | 1.40% |
| Other neuromuscular disorder | 2.00% | 1.70% |
| Other thyroid disorder | 0% | 3.90% |
| Oxygen Deprivation | 0% | 0.60% |
| Parkinson’s Disease | 0% | 0.60% |
| Pelvic Inflammatory Disease | 0.60% | 1.10% |
| Periodic limb movements in sleep | 6.20% | 0.60% |
| Post-traumatic stress disorder | 3.40% | 3.70% |
| Primary dysmenorrhea | 5.30% | 2.20% |
| Psoriasis | 1.70% | 3.90% |
| Reynaud’s phenomenon | 3.40% | 5.10% |
| Rheumatoid arthritis | 1.70% | 4.00% |
| Schizophrenia | 0.30% | 0% |
| Seizures or epilepsy | 0.30% | 2.00% |
| Sensory Loss | 2.80% | 2.80% |
| Sjogren’s syndrome | 0.60% | 2.20% |
| Specific phobia | 6.50% | 0.60% |
| Stroke or TIA | 0% | 1.70% |
| Tourette’s or tic disorder | 0% | 0% |
| Type I Diabetes | 0% | 0.80% |
| Type II Diabetes | 0.30% | 2.80% |

| Supplementary Table 2. Logistic regression analysis for each condition with age at interview (Model 1); repeat size and age at interview (Model 2); and repeat size, repeat size^2^, and age at interview (Model 3). Both Options 1 and 2 are included as affected in models. | | | | | | | | |
| --- | --- | --- | --- | --- | --- | --- | --- | --- |
|  | Model 1: | | Model 2: | | Model 3: | | | |
|  | Age at Interview | | Repeat Size | | Repeat Size | | (Repeat Size)^2^ | |
|  | OR (95% CI) | p-value | OR (95% CI) | p-value | OR (95% CI) | p-value | OR (95% CI) | p-value |
| Anxiety | 0.97 (0.95-0.99) | **<0.001** | 1.00 (0.99-1.01) | 0.689 | 0.95 (0.88-1.02) | 0.178 | 1.00 (1.00-1.00) | 0.158 |
| Depression | 0.99 (0.98-1.01) | 0.541 | 1.00 (0.99-1.01) | 0.746 | 0.99 (0.93-1.06) | 0.837 | 1.00 (1.00-1.00) | 0.792 |
| Migraine Headaches | 0.97 (0.95-0.99) | 0.004 | 1.00 (0.99-1.01) | 0.852 | 0.97 (0.90-1.04) | 0.360 | 1.00 (1.00-1.00) | 0.339 |
| Tension Headaches | 0.98 (0.96-1.00) | 0.027 | 1.00 (0.99-1.01) | 0.794 | 1.00 (0.93-1.07) | 0.983 | 1.00 (1.00-1.00) | 0.982 |
| Sleep Problems | 1.02 (1.00-1.04) | 0.023 | 1.00 (0.99-1.02) | 0.592 | 1.01 (0.94-1.09) | 0.778 | 1.00 (1.00-1.00) | 0.844 |
| Peripheral Neuropathy | 1.01 (0.99-1.03) | 0.367 | 1.02 (1.01-1.03) | **0.001** | 1.11 (1.00-1.22) | 0.039 | 1.00 (1.00-1.00) | 0.106 |
| IBS | 1.00 (0.98-1.02) | 0.760 | 1.00 (0.99-1.02) | 0.450 | 1.03 (0.94-1.12) | 0.509 | 1.00 (1.00-1.00) | 0.584 |
| Osteoporosis | 1.08 (1.05-1.11) | **<0.001** | 0.99 (0.98-1.01) | 0.318 | 1.00 (0.90-1.12) | 0.947 | 1.00 (1.00-1.00) | 0.828 |
| Hypothyroidism | 1.02 (0.99-1.04) | 0.159 | 1.01 (0.99-1.02) | 0.444 | 1.12 (0.99-1.28) | 0.076 | 1.00 (1.00-1.00) | 0.095 |
| Hypertension | 1.07 (1.04-1.10) | **<0.001** | 1.00 (0.99-1.02) | 0.813 | 1.00 (0.91-1.10) | 0.995 | 1.00 (1.00-1.00) | 0.973 |
| RLS | 1.02 (1.00-1.04) | 0.098 | 1.00 (0.97-1.00) | 0.162 | 0.98 (0.89-1.09) | 0.719 | 1.00 (1.00-1.00) | 0.895 |
| Ataxia | 1.04 (1.01-1.06) | 0.003 | 1.01 (1.00-1.03) | 0.054 | 1.03 (0.94-1.13) | 0.537 | 1.00 (1.00-1.00) | 0.757 |
| Sleep Apnea | 1.04 ( 1.01-1.07) | **0.002** | 1.00 (0.98-1.01) | 0.778 | 0.95 (0.87-1.03) | 0.209 | 1.00 (1.00-1.00) | 0.214 |
| Chronic Muscle pain | 1.01 ( 0.98-1.03) | 0.625 | 1.01 (0.99-1.02) | 0.428 | 0.96 (0.88-1.05) | 0.420 | 1.00 (1.00-1.00) | 0.330 |
| Social Phobia | 1.01 (0.99-1.04) | 0.277 | 1.00 (0.98-1.02) | 0.896 | 1.01 (0.91-1.12) | 0.858 | 1.00 (1.00-1.00) | 0.873 |
| Fibromyalgia | 1.00 (0.98-1.03) | 0.698 | 1.01 (1.00-1.03) | 0.125 | 1.01 (0.92-1.11) | 0.824 | 1.00 (1.00-1.00) | 0.972 |
| CFS | 0.99 (0.96-1.02) | 0.490 | 1.01 (1.00-1.03) | 0.083 | 1.04 (0.94-1.15) | 0.488 | 1.00 (1.00-1.00) | 0.667 |
| TMJ | 1.02 (0.99-1.05) | 0.122 | 1.00 (0.99-1.02) | 0.622 | 1.04 (0.92-1.18) | 0.493 | 1.00 (1.00-1.00) | 0.537 |
| OCD | 0.99 (0.96-1.02) | 0.437 | 1.00 (0.99-1.02) | 0.640 | 0.99 (0.90-1.09) | 0.775 | 1.00 (1.00-1.00) | 0.706 |
| ADHD | 0.98 (0.96-1.01) | 0.252 | 1.01 (0.99-1.03) | 0.303 | 0.97 (0.89-1.06) | 0.520 | 1.00 (1.00-1.00) | 0.398 |
| LD | 0.99 (0.96-1.01) | 0.358 | 1.01 (1.00-1.03) | 0.134 | 0.97 (0.89-1.07) | 0.566 | 1.00 (1.00-1.00) | 0.393 |
| Tremor | 1.05 (1.02-1.08) | **<0.001** | 1.01 (0.99-1.03) | 0.208 | 1.04 (0.92-1.18) | 0.488 | 1.00 (1.00-1.00) | 0.610 |
| Bonferroni-adjusted statistical significance p<0.002 are bolded; marginal significance 0.001<p<0.05 are underlined  Abbreviations: IBS: Irritable bowel syndrome; RLS: Restless leg syndrome; CFS: Chronic fatigue syndrome; TMJ: Temporomandibular joint dysfunction; OCD: Obsessive compulsive disorder; ADHD: Attention-deficit/hyperactivity disorder; LD: Learning disability; OR: Odds ratio; CI: Confidence interval | | | | | | | | |

| Supplementary Table 3. Additional information on subjects from FXTAS cluster | | | | | | | |
| --- | --- | --- | --- | --- | --- | --- | --- |
| Subject | Age at Interview | FXTAS Diagnosis | Ataxia | Tremor | Peripheral Neuropathy | Medications | Other reported conditions (Option) |
| 1 | 48 | None | 1 | 0 | 1 | Probiotic | RLS (1); Periodic limb movements in sleep (1) |
| 2 | 67 | Possible | 2  (age 57) | 2  (age 57) | 0 | Amiloride; Indapamide; Finacea; Estrace cream | Osteoporosis (2; age 47) |
| 3 | 57 | None | 1 | 1 | 1 | Estrace cream | RLS (1) |
| 4 | 62 | None (Parkinson’s) | 2  (age 60) | 2  (age 60) | 0 | Selegiline | Osteoporosis (2; age 62); Migraine headaches (2; age 18); Rheumatoid arthritis (2; age 59) |
| 5 | 70 | None | 1 | 1 | 2  (age 70) | Ranexa; Losartan; Metoprolol; Hydrochloro-thiazide; Meloxicam; Clopidrogel; Atorvastatin | Hypertension (2; age 60) |
| 6 | 49 | None | 1 | 0 | 1 | Synthroid | Migraine headaches (2; age 34); Other thyroid: Hashimoto’s (2; age 16); Other autoimmune disorder: Celiac (2; age 47); Depression (2; age 37) |
| 7 | 54 | None | 1 | 1 | 1 | Lisonopril; Alendronate sodium | Osteoporosis (2; age 54); Hypertension (2; age 36); Other autoimmune disorder: alopecia (2; age 54) |
| 8 | 26 | None | 1 | 1 | 1 | None | Hypothyroidism (1); Hyperthyroidism (1); Sleep problems (1); Depression (1); Other phobia (1); PTSD (1); Exposure to hazardous materials (1) |
| 9 | 59 | None | 2  (age 58) | 1 | 1 | Synthroid; Fluticasone proprionate; Omeprazole; Escitalopram; Trilipix | Hypothyroidism (2; age 40); Sleep apnea (2; age 51); Depression (2; age 44) |
| 10 | 57 | None | 1 | 0 | 0 | Synthroid; Premarin; Crestor; Naproxen | Osteoporosis (2; age 50); Migraine headaches (2; age 45); Hypothyroidism (2; age 50); IBS (1); Reynaud’s phenomenon (1); Rheumatoid arthritis (1); Psoriasis (1); Mitral valve prolapse (2; age 40); Social phobia (1) |
| 177 | 63 | None | 1 | 1 | 1 | Losartan; Lovastatin; Fluoxetine; Levothyroxine | Sensory loss (1); Optic neuritis (1); Brain bleed or cerebral aneurism (1); seizures (1); Migraine headaches (1); Muscular dystrophy (2; age 52); Charcot-Marie Tooth disease (2; age 60); Hypothyroidism (2; age 26); Hypertension (2; age 40); Depression (2; age 52) |
| Abbreviations: IBS: Irritable bowel syndrome; RLS: Restless leg syndrome; PTSD: Post-traumatic stress disorder | | | | | | | |
